# “Re-educating” Tumor Associated Macrophages as a Novel Immunotherapy Strategy for Neuroblastoma

**DOI:** 10.3389/fimmu.2020.01947

**Published:** 2020-09-02

**Authors:** Kevin X. Liu, Shweta Joshi

**Affiliations:** ^1^Department of Radiation Oncology, Brigham and Women's Hospital, Harvard Medical School, Boston, MA, United States; ^2^Department of Radiation Oncology, Dana-Farber Cancer Institute, Boston Children's Hospital, Harvard Medical School, Boston, MA, United States; ^3^Division of Pediatric Hematology-Oncology, Department of Pediatrics, UCSD Rady's Children's Hospital, University of California, San Diego, La Jolla, CA, United States

**Keywords:** tumor associated macrophage, neuroblastoma, immunosuppression, polarization, metastasis

## Abstract

Neuroblastoma is the most common extracranial pediatric tumor and often presents with metastatic disease, and patients with high-risk neuroblastoma have survival rates of ~50%. Neuroblastoma tumorigenesis is associated with the infiltration of various types of immune cells, including myeloid derived suppressor cells, tumor associated macrophages (TAMs), and regulatory T cells, which foster tumor growth and harbor immunosuppressive functions. In particular, TAMs predict poor clinical outcomes in neuroblastoma, and among these immune cells, TAMs with an M2 phenotype comprise an immune cell population that promotes tumor metastasis, contributes to immunosuppression, and leads to failure of radiation or checkpoint inhibitor therapy. This review article summarizes the role of macrophages in tumor angiogenesis, metastasis, and immunosuppression in neuroblastoma and discusses the recent advances in “macrophage-targeting strategies” in neuroblastoma with a focus on three aspects: (1) inhibition of macrophage recruitment, (2) targeting macrophage survival, and (3) reprogramming of macrophages into an immunostimulatory phenotype.

## Introduction

Accounting for more than 15% of all childhood cancer deaths, neuroblastoma is a malignant pediatric cancer arising from neural crest cells of the sympathetic nervous system (1, 2). Risk stratification for patients with neuroblastoma have evolved over time, and currently utilize the International Neuroblastoma Risk Group consensus criteria, which includes age at diagnosis, disease stage, tumor pathologic features, including histology and tumor cell ploidy, and tumor genetic characteristics, such as MYCN amplification and chromosomal aberrations (3–6). In particular, MYCN is an oncogenic driver, and MYCN amplification is found in ~40–50% of high-risk neuroblastoma and associated with poor outcomes (7–10).

Treatment regimens for neuroblastoma vary depending on the patient's risk classification. Patients who are diagnosed with very low or low risk neuroblastoma can achieve >90% 5-year overall survival (OS) even after resection alone or observation for asymptomatic patients (3, 5, 11, 12). With response-adjusted chemotherapy and resection, intermediate-risk patients have excellent survival rates of >90% at 5-years (3, 5, 12, 13). Approximately 40–50% of patients are diagnosed with high-risk disease, which requires multimodality treatment, including chemotherapy, surgery, myeloablative chemotherapy with autologous stem cell transplant, radiation therapy, and anti-disialoganglioside (GD2)-based immunotherapy (10, 14–17). For patients with high-risk disease, 5-year OS remains ~50% despite these aggressive strategies (8, 14, 15). Hence, novel effective therapeutic avenues are needed to combat high-risk neuroblastoma.

Immunotherapy in the form of targeted antibodies has revolutionized the field of cancer therapy and is a promising approach to improve outcomes for patients with high-risk neuroblastoma (14). GD2 is a ganglioside that is expressed by neuroblastoma cells and serves as a target for monoclonal antibody (mAb)-based therapeutic intervention. Anti-GD2 mAb therapy is well-tolerated in patients with high-risk neuroblastoma, and a Phase III clinical trial found that the addition of anti-GD2 mAb, interleukin-2 (IL-2), granulocyte-macrophage colony-stimulating factor (GM-CSF) to retinoic acid therapy significantly improved event-free survival (EFS) and OS in high-risk neuroblastoma (18–20). The importance of IL-2 and GM-CSF is unclear with a recent Phase III clinical trial demonstrating no additional benefit to subcutaneous IL-2 (21). Consolidative therapy with anti-GD2 mAb, such as dinutuximab, is now standard of care for patients with high-risk neuroblastoma. Recently, T cells have been engineered to express GD2 chimeric antigen receptor (CAR), and early-phase clinical trials using CAR-T cells for neuroblastoma show this therapy is safe and feasible with some promising results (22–24). However, due to the immunosuppressive tumor microenvironment and lack of tumor antigens (25–28), the approach with CAR-T cell therapy remains challenging in neuroblastoma compared to hematological malignancies. The monoclonal antibodies directed against inhibitory receptors on T cells, such as programmed cell death protein 1 (PD-1) and cytotoxic T-lymphocyte-associated protein 4 (CTLA-4), have shown great efficacy in many adult solid tumors (29, 30). However, in pediatric tumors, these checkpoint inhibitors have shown no significant benefit, which may be due to many factors, including the paucity of neoantigens, development of resistance, and an immunosuppressive environment unique to pediatric solid tumors (31–35). Studies have identified tumor associated macrophages (TAMs) within the immunosuppressive microenvironment of pediatric solid tumors, including neuroblastoma, play important roles in inhibiting both innate and adaptive immune responses (36–39). Hence, a better understanding of the immunosuppressive strategies utilized by macrophages to evade the immune system may help improve responses to immune-directed therapy in pediatric solid malignancies, including neuroblastoma. This review will discuss the neuroblastoma immunosuppressive microenvironment, mechanisms by which TAMs promote tumor progression and immunosuppression in neuroblastoma, and targeting of macrophages as a novel immunotherapy for neuroblastoma.

## Tumor Microenvironment of Neuroblastoma

The tumor microenvironment in neuroblastoma is comprised of cancer associated fibroblasts (CAFs), mesenchymal stromal cells (MSCs), endothelial cells, and immune cells, all of which contribute to promoting a vascular, angiogenic, hypoxic and immunosuppressive milieu around the tumor cells (36, 40–42). Various reports have shown that the microenvironment of neuroblastoma tumors has immunosuppressive components. Defects in antigen presenting machinery (APM) and low levels of MHC class I molecule displayed by neuroblastoma tumor cells lead to decreased cytotoxic T-cell activation and contributes to immunosuppression (43–49). Secretion of different soluble factors, such as transforming growth factor-β (TGF-β), and galectin-1 by tumor cells, directly inhibit T cell function, leading to decreased tumor killing (50–52). The neuroblastoma tumor microenvironment also includes infiltration of various anti-inflammatory immune cells, which inhibits innate and adaptive immune responses and promotes tumor progression (31, 36). In this section, we will discuss the role of immune cells in mediating immunosuppressive microenvironment, and detailed descriptions regarding the role of CAFs, MSCs, and other components in promoting an immunosuppressive tumor microenvironment in neuroblastoma has been reviewed before (36, 40, 42).

**Figure 1:**
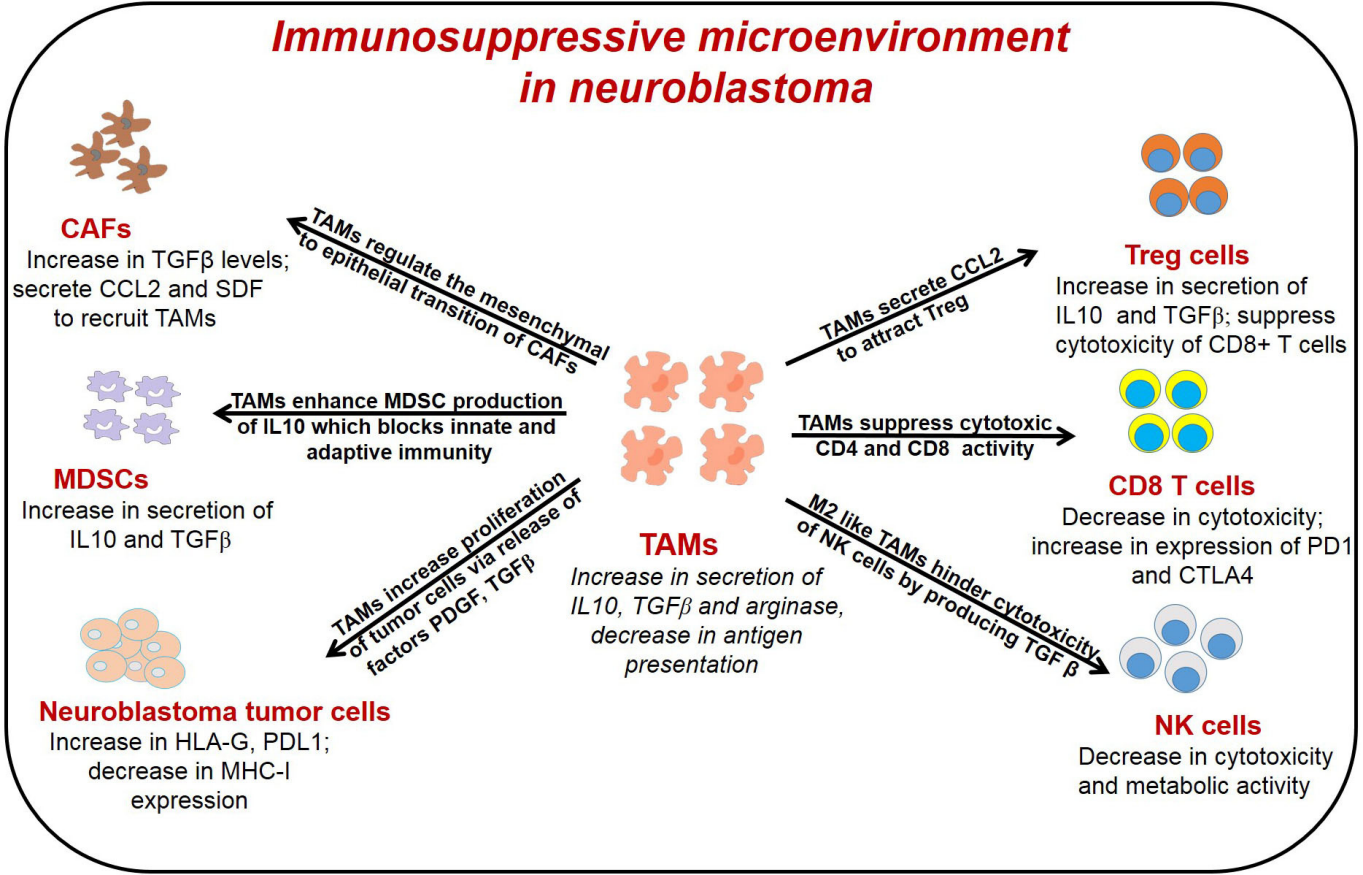
General representation of different immune cells in the TME of neuroblastoma and their interaction with TAMs. TAMs interact with different immune cells to promote tumor angiogenesis, immunosuppression and metastasis. TAMs, tumor associated macrophages; CAFs, cancer associated fibroblast; T reg, regulatory T cells; MDSCs, myeloid derived suppressor cells; NK cells, natural killer cells.

Diverse immune cell populations, including myeloid derived suppressor cells (MDSCs), TAMs, Cluster of differentiation (CD4) CD4+/CD8+ T cells, natural killer (NK) cells, and regulatory T cells (Treg), infiltrate neuroblastoma tumors, and through crosstalk, contribute to immunosuppression. Cancer associated fibroblasts (CAFs), immune cells and tumor cells secrete several cytokines to recruit TAMs and higher infiltration of TAMs in return modulate the functions of other immune cells leading to immunosuppressive microenvironment in neuroblastoma as reported before (36) and illustrated in Figure 1. Early in tumorigenesis, infiltration of cytotoxic CD8+ T cells often predominates; however, as the tumor progresses, the infiltration of TAMs, MDSCs and Treg cells, all of which mediate immunosuppression, increase in the tumor microenvironment (53, 54). In neuroblastoma, presence of cytotoxic CD8+ T cells, CD4+ Th1 cells and NK cells are prognostic factors of improved survival (55). Conversely, the presence of immunosuppressive cells like TAMs, MDSCs, and Treg correlate with poor clinical outcomes in neuroblastoma (25, 56). Importantly, tumor cells and immune cell populations function in concert and through crosstalk to facilitate immunosuppression to further promote tumor growth and metastasis.

In recent years, immune checkpoint blockade has demonstrated that targeting the immune system by activating previously exhausted or dysfunctional T cells can lead to long-term control of various cancers, including melanoma (57–59). In particular, cytotoxic CD8+ T cells are important for tumor cell killing and memory CD8+ T cells are important for durable anti-tumor immunity (60). However, many tumors, including neuroblastoma and other pediatric tumors, do not respond or have limited response to CTLA-4 and PD-1/Programmed death-ligand 1 (PD-L1) blockade (32, 33), and studies have found mechanisms of resistance, including poor infiltration of T cells, tumor cells that lack response to interferon-γ (IFN-γ), and immunosuppressive cell populations in the tumor microenvironment (61). Research has identified many different immune checkpoint receptors and ligands that regulate T-cell function through antigen-presenting cells, and thus, there is growing interest to understand the interactions within the tumor microenvironment between tumor cells, antigen-presenting cells, and T-cells, particularly the contribution of the immunosuppressive tumor microenvironment to tumorigenesis (57, 58).

Within tumors, some myeloid cells may not fully differentiate into dendritic cells, macrophages, or granulocytes, and instead generate a heterogeneous population of immature immunosuppressive myeloid cells, MDSCs (62, 63). In particular, in humans, MDSCs are commonly defined as CD14-CD11b+CD33+ cells that do not have cell-surface markers specific for terminal differentiated myeloid fates and lack expression of HLA-DR (62, 63). In mice, MDSCs are frequently defined by CD11b+Gr1+; however, monocytic and granlocytic subtypes can be further defined by Ly6C^high^/Ly6G- and Ly6C^low^Ly6G+ expression, corresponding to monocytic and granulocytic subtypes, respectively (62). Studies have found that both subtypes of MDSCs along with monocytes and neutrophils are recruited by chemokines, including C-C Motif Chemokine Ligand 2 (CCL2), CCL7, and chemokine (C-X-C motif) ligand 1 (CXCL1), produced by tumor cells and TAMs in neuroblastoma and other tumors (64–67). After their recruitment to the tumor microenvironment, MDSCs play an important role in mediating immunosuppression by inhibiting the activity of T cells, NK cells, and dendritic cells through multiple mechanisms (54, 68, 69). In addition to producing nitric oxide and reactive oxygen species, which directly affect T cell function, MDSCs can deplete L-arginine, a necessary factor for T cell proliferation (66). Moreover, MDSCs produce cytokines, including IL-10 and TGF-β, to induce Treg cells, and inhibit NK cell activation and cytotoxicity (66). Recent studies have also found that MDSCs inhibit dendritic cell differentiation, hinder migration of dendritic cells, and prevent activation of CD8+ T cells (66, 70, 71). Factors, such as hypoxia inducible factor 1α (HIF1α) within the tumor microenvironment also promote differentiation of MDSCs into TAMs, creating a feedback loop to support immunosuppression (66, 72). Studies have shown that targeting MDSCs enhance anti-tumor immune responses in neuroblastoma (73, 74), suggesting that MDSCs play roles in cancer-related inflammation to enhance neuroblastoma tumor progression.

Hence, strategies to block accumulation of MDSCs, recruitment of MDSCs, or polarization of myeloid cells into immunosuppressive MDSCs are under investigation, and molecules targeting these strategies include all-trans retinoic acid (ATRA) and bevacizumab, and have been or are being studied in clinical trials for various cancers including neuroblastoma (27, 39). Interestingly, ATRA and 13-cis-retinoic acid (RA), another retinoic acid, also inhibit neuroblastoma cellular growth and promote differentiation of neuroblastoma cells (75). Phase I clinical trials demonstrated that 13-cis-RA had higher drug levels in patients with high-risk neuroblastoma after bone marrow transplantation (75–77). A subsequent Phase III clinical trial found that 13-cis-RA after bone marrow transplantation for high-risk neuroblastoma improved event-free survival with the most profound effect in patients with minimal residual disease, and 13-cis-RA remains an important component of consolidation therapy for high-risk neuroblastoma (75, 78). A Phase I clinical trial found combination treatment of ATRA and interferon-α2a was well-tolerated in pediatric patients with refractory solid tumors; however, the Phase II clinical trial showed no response in pediatric patients with refractory or recurrent neuroblastoma and Wilms tumors (79, 80). For bevacizumab, one Phase II trial found that no improvement in response rates for bevacizumab in combination with irinotecan and temozolomide in pediatric patients with refractory or recurrent neuroblastoma compared to a historical cohort receiving irinotecan and temozolomide, while preliminary data from another recent Phase II study suggests bevacizumab and a temozolomide-based chemotherapy increased response rates compared to a temozolomide-based chemotherapy alone (81, 82).

Treg cells exhibit their suppressive activity via several mechanisms including inhibition of antigen-presenting cell maturation through the CTLA-4 pathway; secretion of inhibitory cytokines such as IL-10, TGFβ, IL-35; and expression of granzyme and perforin which kills effector T-cells. Preclinical data generated in neuroblastoma mouse models indicates that depletion of Treg cells increases the efficacy of immunotherapy mediated by CD8+ T cells *in vivo* (83–85). The data regarding Treg cells remain less clear in patients with neuroblastoma. Some studies have shown an increased number of circulating Treg cells in patients with neuroblastoma compared to healthy individuals, but was not prognostic of outcomes (86, 87). In another report, lower frequency of Treg cells has been observed in the bone marrow and peripheral blood samples of patients with neuroblastoma compared to healthy controls (88). Interestingly, a higher proportion of Treg cells in the bone marrow and peripheral blood correlated with MYCN amplification (88).

Besides MDSCs and Tregs, TAMs are another cell population that is abundant in the neuroblastoma tumor microenvironment and represent a major driver of tumor immunosuppression in neuroblastoma. The next sections will focus on the role of TAMs in regulating tumor progression and immunosuppression, and the therapeutic potential of targeting TAMs in neuroblastoma.

## TAMs in Neuroblastoma

TAMs are highly infiltrated in the solid tumors and display different phenotypes based on the environmental clues. In this section, we will discuss about the heterogeneity of macrophages, their functions and TAMs as prognostic factor in neuroblastoma.

### Macrophage Heterogeneity

Macrophages are highly heterogeneous immune cells that are primarily phagocytic in nature and involved in host defense and tissue remodeling (89). In response to inflammation and various other environmental stimuli, a plethora of macrophage phenotypes can be induced, which can be generally classified into two main phenotypes based on their gene expression profiles (90). In the presence of lipopolysaccharide (LPS) or IFNγ, macrophages are polarized into classically activated M1 phenotype, producing immunostimulatory cytokines, phagocytosing target cells, and activating adaptive immune responses, whereas M2 polarized macrophages are activated by cytokines, such as IL-4 or IL-13, express scavenger receptors, and secrete vascular endothelial growth factor (VEGF), matrix metalloproteinase 9 (MMP9), IL-10, and TGFβ. Many gene signatures have been identified that differentially associate with M1 and M2 macrophages, with M1 macrophages expressing *Nos2, IL-1*β*, IL-12*β, and *TNF-*α, and M2 expressing *Arg1*, Chitinase 3-like-3 (*Chi3l3*), Resistin-like molecule alpha1 (Retnla/Fizz1), and *CD206* (91–93). Unfortunately, the findings in *in vitro* studies do not always translate to *in vivo* (91–93). M1 and M2 macrophages also have diverse metabolism with M1 relying on glycolysis and expressing nitric oxide synthase and M2 relying on oxidative phosphorylation and expressing arginase (94, 95). Interestingly, data suggests that epigenetic factors also affect polarization of macrophages (96, 97). For example, IL-4 decreased histone H3 lysine-27 (H3K27) methylation at the promoters of M2-associated genes by increasing H3K27 demethylase Jumonji domain containing 3 (Jmjd3) expression in a Signal transducer and activator of transcription 6 (STAT6)-dependent manner (96). These M2 macrophages play an important role in wound healing and tissue remodeling by promoting T helper 2 (Th2) response and dampening immune responses (90, 98).

In recent decades, further subtypes of M2 macrophages, including M2a, M2b, and M2c, have been identified. Stimulated by IL-4 and IL-13, M2a macrophages express cluster of differentiation 206 (CD206) and participate in wound healing by secretion of factors, including TGF-β (99–101). M2b macrophages are induced by immune complexes with toll-like receptor (TLR) or IL-1R agonists, express TNF superfamily member 14 (TNFSF14), and dampen the immune and inflammatory processes in many diseases, including cancer, through release of cytokines, such as IL-10 (99–101). Induced by IL-10 and glucocorticoids, M2c macrophages express Mer receptor tyrosine kinase (MerTK) and also produce factors, such as TGF-β, and cytokines, such as IL-10, to promote tissue remodeling and dampen the immune response (99–101). Interestingly, these M2 subtypes also have distinctive metabolism. M2a and M2c macrophages, but not M2b macrophages, participate in the arginase pathway and utilize glycolysis, while M2b macrophages have increased production of nitric oxide and decreased production of urea (99, 102, 103).

TAMs are macrophages within the tumor microenvironment, often express M2 macrophage markers, such as cluster of differentiation 163 (CD163) or CD206, and secrete VEGF, MMPs, and immunosuppressive cytokines, including IL-10 and TGFβ, all of which dampen effective anti-tumor immune responses and promote tumor progression and metastasis (104). It is important to note that classification of these highly plastic cells as M1 or M2 is an oversimplification, and depending on the signals from tumor microenvironment, these macrophages can easily transition between different activation states fluctuating between M1 and M2 phenotype (98). Furthermore, a recent study demonstrated that tumor hypoxia can control slight variations of gene expression within M2-like TAMs to define a subpopulation of M2-like TAMs with a distinct phenotype to influence angiogenesis (105). Distinct subpopulations of TAMs, including perivascular TAMs and TAMs found at the tumor-stroma interface, have also been described in the tumor microenvironment, and these subsets of TAMs have differential expression of markers with some populations more M2-like and some less M2-like, and contribute to different aspects of tumor progression with perivascular TAMs contributing to metastasis and TAMs at the tumor-stroma interface promoting angiognesis (106–108). Further research is needed to better understand the molecular phenotypes and functional profiles of diverse macrophage subpopulations with the tumor microenvironment and uncover how the interplay between the subsets of TAMs works in concert with other immune cells within the tumor microenvironment and tumor cells to regulate tumorigenesis. Importantly, limitations remain regarding the applicability of preclinical studies of TAMs as these studies often treat TAMs as a single homogeneous population and *in vivo* models may not fully recapitulate the diversity of the human tumor microenvironment, including the heterogeneity of human tumors (109, 110). In addition, studies of TAMs in human tumors are limited by challenges from appropriate macrophage markers to standardization of quantification (111). Given this fluidity of polarization and limitations of current studies, we will discuss the polarization of TAMs throughout the review using the more simplified binary classification of M1 and M2 or M1-like and M2-like.

### TAMs Prognostic Factor in NB

Studies have found that the presence of TAMs correlated with worse prognosis in various solid tumors including neuroblastoma (25, 112). In addition, evidence suggests that TAMs can facilitate progression of neuroblastoma (25, 67). One study used tissue microarrays to assess infiltration of TAMs by CD163 staining in tumor samples from patients with localized disease, stage 4 or metastatic disease, and stage 4S disease (25). Stage 4S is a unique classification for a subset of patients with metastatic neuroblastoma, defined as patients <1 year of age with a localized primary tumor and limited metastases to liver, skin, or bone marrow (<10% involvement), and portends an overall favorable prognosis with 5-year OS of ~90% (25, 113, 114). Stage 4 metastatic tumors had significantly greater numbers of TAMs compared to localized tumors, while there was no significant difference in TAMs observed between Stage 4S and localized tumors (25). Furthermore, the study reported that patients with an age ≥18 months had higher tumor expression of TAM-related genes including *CD14, CD16, CD33, FCGR3, Il-10*, and *IL6R* compared to that of patients with age ≤ 18 months; and age is known to be a prognostic factor and used to guide risk classification (25). Interestingly, a 14-gene signature was found to associate with progression-free survival and TAM-related genes (*CD14, CD16, CD33, FCGR3, Il-10*, and *IL6R*) contribute to 25% of the accuracy of the classification score (25).

A study of 102 non-MYCN-amplified neuroblastoma tumors demonstrated that these tumors express high level of inflammation-related genes expressed by M2 macrophages and identified a gene signature that consists of *IL-6*, IL-6 receptor (*IL-6R), IL-10*, and *TGF*β and is associated with significantly worse prognosis (50). Interestingly, CD68-positive TAMs co-expressing IL-6 was identified in the metastatic bone marrow samples of non-MYCN-amplified neuroblastoma (50). A recent study has shown that the presence of TAMs facilitated up-regulation of MYC protein expression through the signal transducer and activator of transcription 3 (STAT3) pathway in non-MYCN-amplified neuroblastoma tumor cells, suggesting this may at least partially explain the finding that TAMs are associated with poor survival in non-MYCN amplified tumors (67). In comparison, another study analyzing 23 neuroblastoma tumor samples found that stage IV neuroblastoma tumors had higher expression of M1 macrophage markers, IL1-β and TNF-α, while stage I tumors had higher expression of M2 macrophage associated markers IL4, IL10, and TGF-β with IL1-β and TNF-α expression being associated with poor outcomes (115). These differences may be due to sample size, different subtype of neuroblastoma as the former study only included non-MYCN amplified tumors, or the locations of the biopsy samples as tumor microenvironments are often heterogeneous. Furthermore, stage IV tumors that express M1 macrophage markers may form a distinct subgroup of tumors as traditionally TAMs are thought to be M2 polarized and tumors that continue to develop despite a pro-immunostimulatory environment may portend poor outcomes. Taken together, these data suggest that TAMs play an integral role in neuroblastoma tumorigenesis and targeting TAMs may provide a novel treatment avenue in neuroblastoma; however, future studies are needed to clarify the phenotypes of TAMs within the neuroblastoma tumor microenvironment.

### Mechanisms of TAMs in Tumor Progression

TAMs play a pivotal role in primary tumor progression, angiogenesis, metastasis, and immune suppression (116). TAMs interact with tumor cells and other immune cells of the tumor microenvironment to promote these events as shown in Figure 1 and described below.

#### TAMs in Angiogenesis

TAMs secrete various growth factors, including VEGF, Platelet-derived growth factor (PDGF), TGFβ, MMP2, and MMP9, which promote tumor growth by remodeling the extracellular matrix and stimulating neoangiogenesis (117, 118). Hypoxia is also a major contributor of tumor angiogenesis and studies have shown that TAMs are predominantly localized in hypoxic regions of tumors (119). A study by Pietras et al. has shown that HIF2α and CD68-positive TAMs are found in close association with neural crest-like neuroblastoma cells and facilitate angiogenesis in neuroblastoma (120). To promote pro-angiogenic functions within tumors, TAMs up-regulate expression of HIF1α/2α which increases the transcription of various other pro-angiogenic factors like VEGF, TNFα and MMP. Hypoxia also stimulates the entry of TAMs in the tumor microenvironment by secreting several chemokines including CCL2, CCL20, and CSF1 (121, 122). Several studies have shown that CSF1 stimulates macrophages to secrete VEGF to promote angiogenesis (121, 123). Once macrophages are recruited in the tumors, hypoxia-dependent transcription factors HIF1/2α reprograms macrophages into pro-tumoral phenotype that expresses IL-6, VEGF, and inducible nitric oxide synthase (iNOS) and arginase to promote tumor growth, angiogenesis, and immunosuppression (124, 125). While iNOS is typically induced by M1 macrophages, studies have found that M2-like TAMs express iNOS at lower levels than M1 macrophages and these low levels of NO produced by TAMs inhibits tumor cell apoptosis and serves a cytoprotective function (126–128). Recent studies have shown that PTEN/PI3K/AKT signaling axis and Syk-Rac2 signaling axis promotes the stabilization of HIF1/2α in TAMs and polarization of macrophages into immunosuppressive phenotype in solid tumors (129–132). The inhibition of these signaling axes induces degradation of HIF1/2α in a proteasome-dependent manner and suppresses tumor growth and metastasis in solid tumors. The use of dual PI3K/BRD4 inhibitors SF1126 or SF2523 blocks tumor growth, angiogenesis, stabilization of HIF1/2α, and polarization of M2 macrophages in solid tumors including neuroblastoma (131, 133–135).

#### TAMs in Metastasis

TAMs also play an important role in tumor metastasis (104). VEGF and MMPs secreted by TAMs not only promote tumor angiogenesis, but also increase the permeability of blood vessels to facilitate extravasation. TAMs promote both the release of metastatic tumor cells from their primary site and establishment of tumor cells at secondary distant sites. In neuroblastoma, area of cancer-associated fibroblasts (CAFs) in the tumor microenvironment correlates with higher disease stage and higher risk stratification, and associated with increased TAMs (65). CAFs, TAMs, and tumor cells participate in crosstalk with neuroblastoma cells promoting activation of CAFs from bone marrow-derived mesenchymal stem cells and TAMs inducing increased invasion of CAFs, while CAFs can induce further neuroblastoma cell proliferation and inducing invasion of TAMs (65, 136). Moreover, CAFs release stromal cell-derived factor 1 (SDF1)/ C-X-C motif chemokine 12 (CXCL12) to recruit TAMs and endothelial progenitor cells to promote angiogenesis (137). CAFs also secrete TGFβ to facilitate epithelial to mesenchymal transition and in turn, TAMs promote mesenchymal to epithelial transition to enhance reactivity of CAFs (138, 139). Hashimoto et al. has shown that TAMs have increased expression of CXCL2 which enhances neuroblastoma tumor invasion through CXCL2/CXCR2 signaling (65). Our studies have shown that PTEN/PI3K signaling axis in macrophages promotes tumor metastasis and use of dual PI3K/BRD4 inhibitors SF1126 or SF2523 blocked tumor metastasis in neuroblastoma mouse model (134, 135).

#### TAMs in Immunosuppression

TAMs can promote immunosuppression in the tumor microenvironment by (1) suppressing the cytotoxic and metabolic activity of NK cells; (2) expanding Treg cells which indirectly suppress the function of effector T cells; (3) interacting with cytotoxic T cells in antigen-specific and antigen non-specific manners; and (4) stimulating MDSCs to secrete IL-10 which inhibits innate and adaptive immune responses.

The efficacy of anti-GD2 targeted immunotherapy in neuroblastoma relies on the antibody-dependent cellular cytotoxicity (ADCC) mediated by natural killer cells. Studies have shown that TAMs suppress the activity of NK cells in neuroblastoma (140). Song et al. found that high-risk neuroblastoma tumors contain CD68+ TAMs that secrete IL6 to suppress cytotoxic activity of NK cells (51). Xu et al. demonstrated co-culture of neuroblastoma tumor cells with macrophages leads to secretion of IL-6 and TGFβ, which inhibits cytotoxicity of IL-2 activated NK cells (140). Lenalidomide is an analog of thalidomide and has broad functions in diverse cancers, including direct anti-tumor activity, anti-angiogenic effects, and immunomodulatory effects (141). In terms of immunomodulating properties, lenalidomide induces T-cell activation and induces IL-2, IFN-γ, and TNF-α expression without requiring co-stimulation, while blocking IL-6 and TGFβ secretion, which contribute to dysfunction of immune cells, including T-cells and dendritic cells (142). Lenalidomide has been extensively studied in multiple myeloma and hematologic malignancies (142); however, lenalidomide also enhances the activation of NK cells and improves survival in a xenograft model of neuroblastoma (140). In another study, Liu et al. showed that CCL20-producing TAMs generate a hypoxic trap for tumor-infiltrating NK T cells and IL-15 can protect antigen-activated NK T cells from hypoxia and immunosuppressive effects of TAMs (143).

In addition to regulating function of NK cells, TAMs also suppress the activation of CD8+ T cells by (1) generating anti-inflammatory cytokines which inhibits function of T cells, (2) depleting metabolites required for T cell proliferation, and (3) activating T cell checkpoint blockade through engagement of T cell receptors (39, 104, 144). TAM-derived enzymes arginase 1 and indoleamine dioxygenase 1/2 (IDO 1/2) catalyze metabolism of L-arginine and L-tryptophan respectively, and leads to suppression of effector T cell activation (110). A recent study showed that macrophage-derived IL-1 and TNF-α regulated arginine metabolism in neuroblastoma cells through a signaling pathway dependent on RAC-alpha serine/threonine-protein kinase (AKT) (115). Furthermore, *in vitro* studies suggest that neuroblastoma tumor cells can also promote M1 polarization of TAMs, leading to immune-metabolic cross-talk and a feedback loop that promotes neuroblastoma progression through tumor growth and immunosuppression (115); however, these findings have not yet been explored *in vivo*. Moreover, M2 macrophages preferentially promote oxidative phosphorylation and fatty acid oxidation over glycolysis, and increase availability of glucose for tumor cells (145). These changes in metabolism also supports Treg cells, which utilize oxidative phosphorylation, and inhibits CD4+ T cells, CD8+ T cells, and NK cells, which depend heavily on glycolysis (145–147). TAMs can also inhibit function of effector T cells by expressing ligands of inhibitory receptors PD-1 and CTLA-4. TAMs also up-regulate expression of Programmed death-ligand 1 (PD-L1), under the influence of HIF1α, leading to suppression of T cell activity in hypoxic tumor regions (148). PD-L1 has also been found to promote glycolysis in tumor cells that also compete for glucose with tumor-infiltrating lymphocytes, which also contributes to tumorigenesis (149). A recent study by Mao et al. found that myeloid cells isolated from neuroblastoma tumors express high levels of PDL1, and combining CSF1R inhibition with anti-PD1/PDL1 antibodies lead to better anti-tumor immune response and improved survival in mouse model of neuroblastoma (56). These preclinical data suggest that targeting TAMs either alone or in combination with checkpoint inhibitors may provide a novel treatment avenue in neuroblastoma and warrants further clinical investigation.

## TAM Targeted Immunotherapy in Neuroblastoma

**Figure 2:**
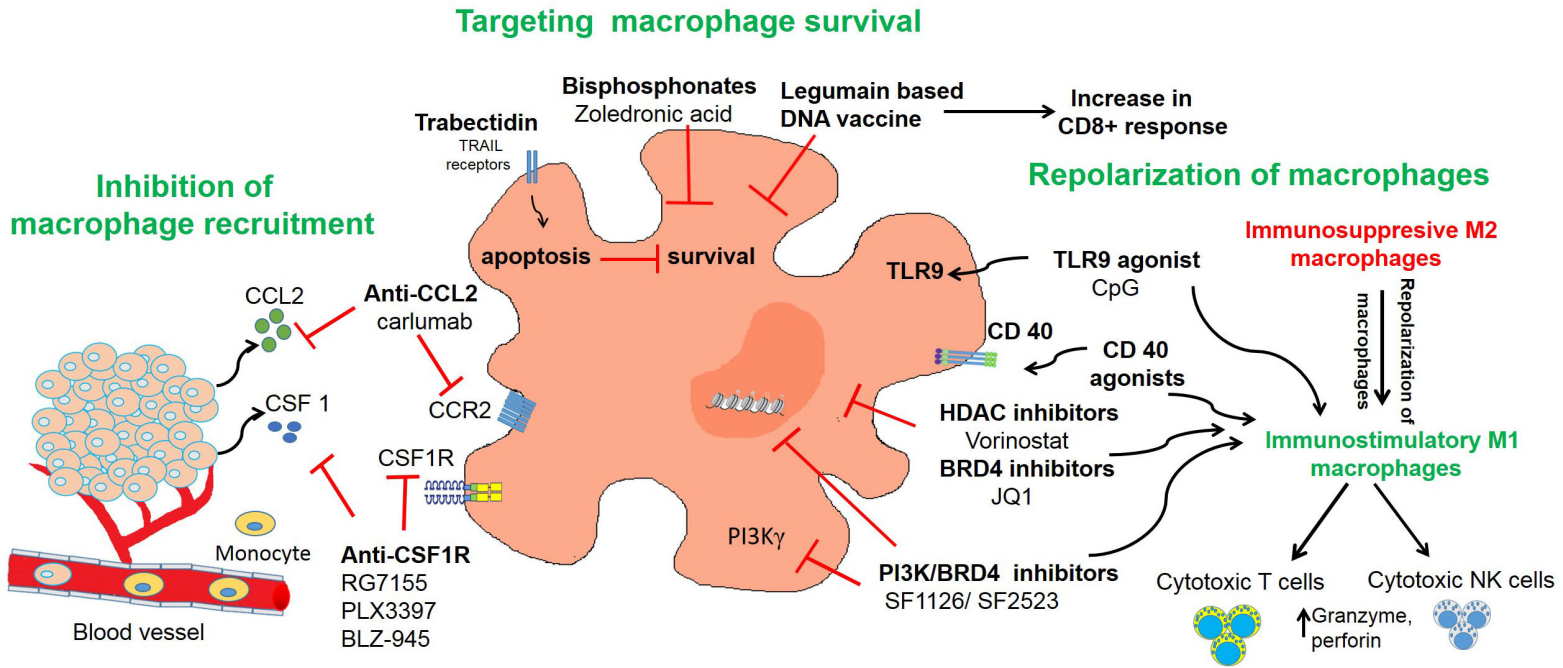
Targeting of macrophages as an effective strategy to improve anti-tumor immune responses in neuroblastoma. The figure illustrates three different strategies used in neuroblastoma to inhibit recruitment of macrophages or to deplete TAMs or to repolarize M2 macrophages into M1 macrophages.

Given the abundant infiltration of macrophages in the tumor microenvironment and their diverse functions in promoting tumor growth, angiogenesis, metastasis, and immune escape, studies have found that TAMs may serve as an important target for immunotherapeutic strategies (150). TAM re-education has been proposed as an effective strategy to treat cancer. In particular, targeting this population has gained significant attention as this immune cell bears potential to reverse immunosuppression and synergize with checkpoint inhibitors or radiotherapy to activate cytotoxic T cells to kill tumors. To overcome the immunosuppressive functions of TAMs in neuroblastoma, current therapeutic strategies are aimed on three aspects as shown in Table 1 and Figure 2 and described in following sections:

**Table 1:** Therapeutic approaches to target TAMs in neuroblastoma.

**Mechanism of action**	**Target**	**Treatment strategy**	**Macrophage markers**	***In vivo* model**	**Outcome**	**References**
Inhibition of macrophage recruitment	CSF1	siRNA knockdown of CSF-1	F4/80^+^, Tie-2^+^	SK-N-DZ athymic nu/nu (nude) xenograft mouse model	Decreased TAMs; decreased tumor growth; increased survival	(121)
	CSF1R	BLZ-945 + anti-CSF-1 mAbs (MCS110 and 5A1) + cyclophosphamide + topotecan	Ly6G^−^ CD11b^+^F4/80^+^	CHLA-255-Fluc NOD/SCID xenograft mouse model	Decreased TAMs; decreased tumor growth; increased survival	(122)
		BLZ-945 + anti-PD1	CD11b^+^F4/80^+^	TH-MYCN transgenic mouse model	Decreased TAMs, decreased tumor growth	(56)
Intervention with TAM survival	Osteoclasts	Ibandronate/ bisphosphonate	TRAP^+^	SK-N-BE athymic nude xenograft mouse model	Decreased number of osteolytic lesions	(151)
	Legumain	LEG3	CD68^+^	C1300 A/J xenograft mouse model	Decreased tumor growth; Increased survival	(152)
	TRAIL receptors	Trabectidin + cisplatin	F4/80+	SK-N-DX athymic nude xenograft mouse model	Decreased tumor growth	(153, 154)
Repolarization of macrophages	Rac2	Genetic deletion of *Rac2*	F4/80^+^	9464D-GD2 syngeneic mouse model	Decreased tumor growth and polarization of M2 macrophages	(130)
	BRD4	JQ1	F4/80+	BE(2)-C-LucNeo NOD/SCID xenograft mouse model; SFNB-06 athymic nude xenograft mouse model; TH-MYCN transgenic mouse model	Decreased tumor growth; increased survival; Blocked polarization of M2 macrophages *in vitro*	(134, 135, 155, 156)
	HDAC	Vorinostat	CD11c^dim^F4/80^high^ MHCII^int^	TH-MYCN transgenic mouse model	Increased expression of *CD163, IL4Ra, FcRg1, FcRg3, FcRg4*; Decreased expression of *FIZZ1, YM1, Arginase*; Decreased tumor growth	(157)
	PI3K/BRD4	SF2523	CD11b^+^F4/80^+^ CD206^+^	SKNBE2 athymic nu/nu (nude) xenograft mouse model	Suppressed polarization of M2 macrophages *in vitro*; Decreased tumor growth	(134, 135)
	JAK1/2	Ruxolitinib	CD11b^+^F4/80^+^	NBT2 NOD/SCID xenograft mouse model	No evaluation of TAM-associated markers *in vivo*; Decreased tumor growth	(67, 89)
	CD40	Anti-CD40 +CpG	CD11b^+^F4/80^+^	NSX2 NOD/SCID xenograft mouse model	No evaluation of TAM-associated markers *in vivo*; Decreased tumor growth; Increased survival	(158, 159)
		Anti-CD40+ CpG + anti-CTLA-4	Ly6G^−^CD11b^+^	9464D-GD2 syngeneic mouse model	No evaluation of TAM-associated markers *in vivo*; decreased tumor growth	(159, 160)

### Inhibition of Macrophage Recruitment

Inhibition of macrophage recruitment has shown great efficacy in blocking tumor growth and metastasis in various solid tumors including neuroblastoma (56, 122). Chemokines secreted by tumor cells, such as CCL2 and CSF1, recruit and polarize monocytes (161). One promising target, CSF-1 receptor (CSF1-R) is exclusively expressed on normal monocytes and plays a crucial role in regulating macrophage survival. Recent evidence suggests that CSF1-R infiltrating myeloid cells or monocytes correlates with poor clinical outcome in patients with neuroblastoma (56). One study found that CSF-1 blockade decreased neuroblastoma tumor growth *in vivo* and prolonged survival in the SK-NDZ neuroblastoma xenograft model, in which tumor cells do not express human CSF-1 (121). Antagonists and antibodies to CSF1R has been developed and tested in various preclinical mouse models. RG7155 (Emactuzumab) is a humanized monoclonal antibody that blocks CSF-1R activation and shows efficacy in mouse tumor models of colon cancer and fibrosarcoma, and a phase 1 clinical trial showed a partial response with emactuzumab in five of seven patients with diffuse-type tenosynovial giant cell tumors (162, 163). Another phase I trial studied emactuzumab with or without paclitaxel, in adult patients with advanced solid tumors and found that both monotherapy and combination with paclitaxel was largely safe with only one grade 5 toxicity, decreased CSF-1R+ and CD68+/CD163+ macrophages in the tumor microenvironment, and achieved a 7% response rate in patients receiving combination therapy with three patients with breast cancer and one patient with ovarian cancer achieving a partial response (164). PLX3397 (Pexidartinib) is a CSF1-R inhibitor and a phase II trial demonstrated efficacy of pexidartinib in patients with tenosynovial giant cell tumor with a 39% response rate compared to 0% with placebo (NCT02371369) (165). Various clinical trials of this compound either alone or in combination with radiotherapy are under investigation for glioblastoma and other solid tumors (166). Another CSF-1R inhibitor, BLZ-945 blocked glioma progression and improved survival in various preclinical models (167).

Mao et al. reported that BLZ-945 modulates immunosuppressive myeloid cells and suppresses tumor progression in TH-MYCN mouse model of neuroblastoma (56). Interestingly, BLZ-945 in combination with anti-PD-1 antibody showed synergy in improving the survival of TH-MYCN mouse model of neuroblastoma with noted greater T-cell activation and infiltration into the tumor microenvironment (56). Depletion of TAMs in neuroblastoma mouse models potentiates the efficacy of chemotherapy even in the absence of T lymphocytes (122). BLZ-945 in combination with cyclophosphamide and topotecan inhibited neuroblastoma growth and improved survival in subcutaneous and intra-renal neuroblastoma tumors in immunodeficient NSG or NOD/SCID mice, suggesting that CSF-1R blockade with chemotherapy might be effective in patients with neuroblastoma and restricted anti-tumor T cell responses (122). This exciting data provides preclinical evidence that CSF1R inhibitors have therapeutic potential alone or in combination with chemotherapy or immunotherapy in neuroblastoma, and warrants further clinical investigation.

CCL2 is also an important chemokine secreted by tumor cells and endothelial cells to support infiltration of TAMs in tumors (168). Blockade of either CCL2 or its receptor CCR2 has shown anti-tumor activity in various preclinical models (169). In the Phase I clinical trial, administration of neutralizing antibodies against CCL2 (carlumab, CNTO888), was well tolerated and showed clinical reponse in patients with solid tumors; however, there was no response in the Phase II clinical trial for patients with castration-resistant prostate cancer (170, 171). Interestingly, sphingosine-1-phosphate (S1P) has been shown to be an important regulator of CCL2 expression in neuroblastoma, and preclinical studies suggest that inhibition of S1P2 leads to decreased CCL2 signaling and decreased macrophage infiltration in neuroblastoma tumors along with antitumor activity *in vivo* (172, 173).

### Targeting Macrophage Survival

Strategies targeting the survival of macrophages are also under investigation for various solid tumors (56). Trabectidin (ET-743) is an anti-tumor agent recently shown to deplete circulating monocytes and TAMs in cancer patients (154, 174). Trabectidin activates caspase-8 dependent apoptosis in mononuclear phagocytes and shows anti-tumor activity mainly due to its cytotoxic effects on TAMs. Trabectedin-induced TAM reduction was associated with decreased angiogenesis in murine tumors and human sarcomas (154). The combination of cisplatin and trabectidin has shown anti-tumor activity in neuroblastoma xenografts (153). Although this study didn't evaluate if administration of trabectidin can deplete monocytes, its findings suggest that trabectedin has activity in neuroblastoma and future studies should explore whether this drug can deplete macrophages to enhance anti-tumor immune responses in neuroblastoma.

Ongoing research has also examined the role of using activated NK cells to deplete the monocyte or macrophage population. CD1d is a MHC class I-related protein that is used by antigen-presenting cells to present lipid and glycolipid antigens that are subsequently recognized by NK T cells and leads to NK T cell activation (175, 176). Traditionally, NK T-cell therapy has been used to target tumor cells, and while neuroblastoma tumor cells do not express CD1d, studies have found that inducing exogenous CD1d expression in neuroblastoma tumor cells with pulsed α-Galactosylceramide activated NK T cells and produced NK T cell anti-tumor activity (177). Interestingly, TAMs in the neuroblastoma tumor microenvironment are CD1d-positive, and transferring activated NK T cells led to increased cell death of TAMs in a CD1d-restricted manner and decreased tumor proliferation *in vivo* (51). Interestingly, NK T cell anti-tumor activity was not observed for *in vivo* models using tumors grown in the absence of monocytes, suggesting that the decreased tumor growth is specific to NK T cell killing of TAMs (51). Furthermore, CD105 is a transmembrane coreceptor for TGFβ and bone morphogenetic protein-9 (BMP-9) and many immune cell populations within the tumor microenvironment, including MSCs, CAFs, proliferating endothelial cells, and TAMs express CD105 (178–182). Use of CD105 antibodies to deplete CD105-positive immune cells, including macrophages, from the tumor microenvironment in addition to dinutuximab and activated NK cells decreased tumor growth and improved survival in murine models of neuroblastoma as well as a patient xenograft model compared to other combinations of therapy (182).

Some other treatment strategies in neuroblastoma that decrease macrophage survival also have anti-tumor activity. Bisphosphonates have long been used for the treatment of osteoporosis and osteolytic lesions by bone metastasis in neuroblastoma (151, 183–185). Recent studies have shown that bisphosphonates have anti-tumor activity and are cytotoxic against myeloid cells, tissue macrophages, and TAMs (186). Vorotnjak et al. has evaluated the anti-tumor effect of bisphosphonates on neuroblastoma cells and found that nitrogen-containing bisphosphonates are more cytotoxic than non-nitrogen containing bisphosphonates (187). Sohara et al. found that osteoclasts (bone macrophages) contribute to neuroblastoma bone metastases and demonstrated that treatment of mice engrafted with neuroblastoma tumors with bisphosphonates can delay the progression of osteolytic lesions (151). Zoledronic acid is a nitrogen-containing bisphosphonate that has been used for the treatment of bone metastasis in solid tumors (188). Peng et al. has shown that zoledronic acid inhibits the activity of osteoclasts within neuroblastoma bone lesions, but also blocks the proliferation and survival of tumor cells, and in combination with cytotoxic chemotherapy prolongs survival of mouse model of neuroblastoma with bone invasion (183). On the basis of these preclinical studies, a phase I clinical trial evaluated the efficacy of zoledronic acid with low-dose cyclophosphamide in patients with refractory neuroblastoma. The results of this phase I trial suggest that this combination was well-tolerated and decreased osteoclast activity and serum IL-6 levels in patients with relapsed or refractory neuroblastoma (185). A subsequent Phase I study from the New Approaches to Neuroblastoma Therapy Consortium (NCT00885326) explored the safety of bevacizumab, cyclophosphamide, and zoledronic acid in patients with recurrent or refractory high-risk neuroblastoma with a secondary aim of tumor response, and while it has completed accrual, preliminary data is not yet available. Future research is needed to assess the contribution of osteoclasts and TAMs in metastatic neuroblastoma and whether the efficacy of bisphosphonates in neuroblastoma is dependent on its effect on osteoclasts, anti-tumor activity, or both.

Furthermore, strategies aimed at targeting cell surface proteins of M2 macrophages is also under investigation in preclinical models. Legumain is a promising target as it is expressed on CD206^+^/F4/80^+^ TAMs and a legumain-based DNA vaccine induced a robust CD8+ T cell response against TAMs in murine models of metastatic breast cancer, colon cancer and non-small cell lung cancer (189). In the syngeneic C1300 neuroblastoma tumor model, legumain-activated doxorubicin prodrug LEG3 has anti-tumor activity (152). While Wu et al. did not explore the effect of legumain on the tumor microenvironment and TAMs, the syngeneic neuroblastoma tumor model has an intact immune system, it is possible effects on the tumor microenvironment may have contributed to the decrease in tumor growth and improved survival. Thus, future studies are need to understand the role of legumain on regulating immune cell populations, particularly, TAMs, in neuroblastoma. Given the crosstalk between tumor cells and immune cell populations, novel treatments may need to target multiple pathways, and the strategy of eliminating TAMs either alone or in conjunction with agents showing anti-tumor activity still needs further investigation in neuroblastoma.

### Repolarization of Macrophages

Macrophage repolarization is another cancer immunotherapy strategy under investigation as fine-tuning their functional plasticity can transition pro-tumor macrophages into anti-tumor M1 macrophages (190). Agents that can polarize macrophages into anti-tumorigenic phenotype can be beneficial in cancer therapy. M2-like TAMs inhibit the activation of CD8+ T cells and NK cells and repolarization of macrophages into M1 phenotype activates CD8+ T cells and NK cells (Figure 2). One strategy to repolarize macrophages *in vivo* is the inhibition of Syk-Rac2 signaling axis. Our group has identified a novel signaling pathway by which α_4_β_1_ integrin and CSF receptor activates Syk-PI3Kγ-Rac2 axes to polarize macrophages into immunosuppressive phenotype (130–132). Rac2 is a hematopoietic GTPase and our studies have shown that macrophage deletion of Rac2 blocks tumor growth in syngeneic NB9464 model of neuroblastoma (130). Moreover, genetic deletion or pharmacological inhibition of macrophage Syk kinase induces proinflammatory transcriptional programming in macrophages which resulted in activated T cell responses in lung adenocarcinoma and B16 melanoma model (132). These studies also identified that Syk kinase regulates stabilization of HIF1/2α to promote immunosuppression in TME while inhibition of Syk kinase promotes activation of NF-κB, leading to immunostimulation and tumor regression. PI3Kγ is another molecular target that is highly expressed in macrophages and promotes anti-inflammatory polarization of macrophages in solid tumors (131, 135). Our recent study demonstrated that combinatorial inhibition of Syk-PI3Kγ axis by a dual Syk-PI3Kγ inhibitor SRX3207 has shown great efficacy in solid tumors (132). Other strategies to repolarize macrophages include targeting histone deacetylase (HDAC) proteins or epigenetic reader proteins, including bromodomain-containing proteins (BRD). We have recently shown that BRD4 promotes polarization of macrophages into anti-inflammatory phenotype and treatment with bromodomain inhibitor JQ1 or dual PI3K/BRD4 inhibitor SF1126 or SF2523 blocks immunosuppression and promotes adaptive immune responses in solid tumors including neuroblastoma (131, 134, 135, 155, 156). Vorinostat, a histone deacetylase inhibitor, also promotes M1 repolarization of TAMs, along with increased GD2 expression on neuroblastoma cells with combination of vorinostat and anti-GD2 immunotherapy showing synergy *in vivo* (157).

Another target to repolarize macrophages is Janus kinase 2 (JAK2)/STAT3 pathways. STAT3 is activated in myeloid cells and promote tumor immunosuppression (191). The presence of TAMs upregulated the activation of STAT3 pathway in neuroblastoma tumor cells and administration of JAK/STAT inhibitor AZD1480 reduced TAM-mediated growth of neuroblastoma (67).

In recent years, emerging evidence shows that CD40 plays a critical role in regulating antitumor effector macrophages with M1 polarization, leading to production of IFN-γ, IL-12, and nitric oxide (NO) to help mediate tumor killing (192, 193). Agonistic anti-CD40 antibodies induce tumoricidal effects against neuroblastoma cells *in vivo* (192, 194, 195). Buhtoiarov et al. has shown that anti-CD40 antibody in combination with CpG-containing oligodeoxynucleotides (CpG), a toll-like receptor 9 agonist led to activation of tumoristatic macrophages and promoted anti-tumor immunity in NXS2 mouse model of neuroblastoma model (158). Voeller et al. studied two syngeneic murine models of neuroblastoma, NXS2, and N-MYC driven 9464D-GD2, which have moderate and low tumor mutational burden (TMB), respectively. Radiation and treatment with immunocytokine hu14.18-IL2, a fusion protein linking hu14.18 anti-GD2 mAb and IL2, produced significant anti-tumor response in NXS2 mice, but not in N-MYC driven 9464D-GD2 mice, compared to monotherapy. While the addition of CTLA-4 inhibition alone was not effective in N-MYC driven 9464D-GD2 mice, treatment with CpG, and anti-CD40 in addition to anti-CTLA-4, radiation, and the immunocytokine produced complete tumor regression in four of five mice, and increased macrophages and decreased Treg cells within the tumor microenvironment. On rechallenge experiments, tumors grew significantly slower in previously treated mice compared to treatment-naïve mice, suggesting a memory response (160).

Other macrophage repolarization strategies have also been explored for neuroblastoma. Relation et al. demonstrated that MSC-associated delivery of IFN-γ directly to the tumor microenvironment causes M1 polarization of TAMs, leading to significantly decreased tumor growth and increased survival in a model of metastatic neuroblastoma (196). In addition, prostaglandin E_2_ (PGE_2_) promotes neuroblastoma tumor growth through multiple pathways, including facilitating M2 macrophage polarization, and inhibition of PGE2 leads to repolarization of macrophages to a M1 state and reduced tumor growth in TH-MYCN transgenic mice (197). Finally, delivery of an oncolytic virus containing an antagonist of C-X-C motif chemokine receptor 4 (CXCR4) alters the tumor microenvironment with decreased Treg cells and increased proportion of immunostimulatory macrophages (198).

## Limitations of *in vivo* Preclinical Models of Neuroblastoma to study Tams

Preclinical models play an important role in drug discovery for many cancers, including neuroblastoma, but each model is not without its limitations and likely contribute to the high failure rate of promising preclinical targets in clinical trials (199–203). Xenograft, transgenic, and syngeneic neuroblastoma mouse models are frequently used to characterize the role of TAMs in neuroblastoma and investigate novel therapeutic avenues. Established neuroblastoma cell lines are often used as they have been heavily characterized, but due to selection in an *in vitro* environment, these cell lines may acquire additional genetic alterations that are distinct from neuroblastoma tumors and influence the results generated (203). Patient-derived xenografts can be excellent models because they are derived from a patient's tumor sample and therefore recapitulate molecular and phenotypic features that resemble neuroblastoma tumors (203). Moveover, xenograft models are generated by using immunodeficient mice, such as athymic nude mice, which carry loss of *forkheadbox n1* (*Foxn1*), and severely compromised immunodeficient (SCID) mice (200, 202, 203). Atymic nude mice lack mature T cells, but continue to have myeloid cells and have innate immunity pathways, which often makes these mice, an excellent *in vivo* model to study TAMs and MDSCs (204). Nonetheless, crosstalk and interplay between the diverse immune populations, including T cells and TAMs, are important in regulating neuroblastoma tumor growth. Thus, these models have a compromised immune system that does not completely recapitulate the complex tumor microenvironment in patients with neuroblastoma.

There are several transgenic models of neuroblastoma, including the widely used TH-MYCN neuroblastoma model (205–208). The TH-MYCN neuroblastoma model overexpressing MYCN in neuroectodermal cells through the use of a tyrosine hydroxylase promoter; however, the incidence of spontaneous neuroblastoma varies between 5 and 20% in C57Bl6/N background to 40% in the 129X1/SvJ background, suggesting other genetic factors may contribute to the development of neuroblastoma (205, 207). Recently, a Cre-conditional mouse model of neuroblastoma (LSL-MYCN;Dbh-iCre), and induces conditional expression of MYCN in dopamine β-hydroxylase-expressing cells through Cre recombination and carries ~75% incidence of spontaneous tumor development regardless of strain background (208). Mutation in anaplastic lymphoma kinase (ALK) have been recently found it a subset of patients with neuroblastoma and a transgenic mouse model expressing ALK^F1174L^ in neural stem cells was generated using either Cre recombination in either dopamine β-hydroxylase- or tyrosine hydroxylase-expressing cells (206). While these mice have an intact immune system, which is excellent for studying the tumor microenvironment, these models depend on MYCN over-expression or ALK^F1174L^ targeted expression, which only captures a subset of neuroblastoma cases, and may not be representative for non-MYCN amplified and non-ALK mutated disease. Little is currently known about the differences in tumor microenvironment for tumors with different oncogenic driver mutations and it is possible the immune response is different between each of these models. Furthermore, the TH-MYCN and ALK^F1174L^ transgenic mouse models have a low incidence of bone metastases (205, 206), suggesting that tumors from patients with high-risk neuroblastoma, in whom bone metastases are common, may be different from those that develop in these transgenic models.

Syngeneic mouse models are generated by using neuroblastoma cell lines that were originally derived from genetically engineered models to generated allograft mouse models of neuroblastoma (209). Unlike transgenic mouse models, the mutations are only present in the tumor cells that are used to generate the allograft model, thereby limiting any potential off target effects, these cell lines can be modified to have additional mutations or improve imaging or tracking of tumors, and these mice have a fully functioning immune system, which can better mimic the tumor and tumor microenvironment (209). However, similar to xenograft models, the cell lines may have genetic alterations as they are grown *in vitro*, and similar to transgenic models, these cell lines will only carry the specific mutations introduced in the genetically engineered mouse models. Furthermore, unlike patient xenografts, some of the tumor heterogeneity may not be fully recapitulated (199, 209).

Many preclinical *in vivo* models can be used to study TAMs and the tumor microenvironment and many studies have identified novel treatment strategies that warrant further evaluation in clinical studies for patients with neuroblastoma (Table 1). Although each type of model has various advantages and disadvantages, these studies continue to provide a better characterization of the tumor microenvironment within neuroblastoma and further understanding of novel pathways that are integral to neuroblastoma tumor progression. Due to the limitations of these models, it is possible that the treatment strategies identified within these preclinical studies may benefit a subset of patients with neuroblastoma and patient selection may be important for future clinical investigations.

## Conclusion

While the tumor microenvironment in neuroblastoma is complex and are comprised of many players, including CAFs, TAMs, T-cells and other immune cells, increasing evidence suggest that TAMs are central regulators of tumor progression and contribute to tumor immunosuppression, and serve as a novel target for future treatment strategies in neuroblastoma. Novel therapeutic agents blocking TAM recruitment, depleting macrophages, or repolarizing macrophages to M1 states have been under investigation in preclinical models of neuroblastoma with promising outcomes. It remains important to recognize that these novel strategies have other effects on the tumor microenvironment beyond TAMs and may also have anti-tumor activity. Given the complex cross-talk within the tumor microenvironment between tumor cells and immune cell populations, drug targets or immunotherapies that target multiple pathogenic pathways may be important. Future studies will further delineate signaling mechanisms utilized by these innate immune cells to promote immunosuppression and identify novel treatment strategies to re-activate the tumor immune response and overcome checkpoint blockade in neuroblastoma.

## Author Contributions

KL wrote and reviewed the manuscript. SJ wrote, reviewed, and proofread the manuscript. Both authors contributed to the article and approved the submitted version.

## Conflict of Interest

The authors declare that the research was conducted in the absence of any commercial or financial relationships that could be construed as a potential conflict of interest.
